# Field Epidemiology Training Programs (FETP) in the Eastern Mediterranean Region and Bangladesh: graduates’ skills application, career advancement, and continuing education needs

**DOI:** 10.3389/fpubh.2025.1669324

**Published:** 2026-01-12

**Authors:** Yousef Khader, Mohannad Al Nsour, Sara Abu Khudair, Ali Ahmed Al-Waleedi, Rana AlHamawi, Haitham Bashier, Ruba Kamal Alsouri, Amna Khairy, Ayman Bani Mousa, Amal Al-Maani, Abdulla Salem Bin-Ghouth

**Affiliations:** 1 Department of Public Health, Jordan University of Science and Technology, Irbid, Jordan; 2 Eastern Mediterranean Public Health Network, Amman, Jordan; 3 Department of Community Medicine and Public Health, Aden University, Aden, Yemen; 4 Qatar Field Epidemiology Training Program, Ministry of Public Health, Doha, Qatar; 5 Epidemic Administration Directorate, Jordan Ministry of Health, Amman, Jordan; 6 Center for Disease Control and Prevention, Ministry of Health, Muscat, Oman; 7 Faculty of Medicine and Health Sciences, Hadhramaut University, Al Mukalla, Yemen

**Keywords:** Field Epidemiology Training Program, career path, epidemiological skills, continuing education, skills

## Abstract

**Introduction:**

This study aimed to assess the utilization of field epidemiology skills and the perceived confidence of graduates in applying these skills throughout their careers. It also sought to assess the impact of the Field Epidemiology Training Program (FETP) on professional growth across different tiers, identify continuing education needs, and identify areas of dissatisfaction.

**Methods:**

This cross-sectional study surveyed FETP graduates from various countries in the Eastern Mediterranean Region (EMR) and Bangladesh. An online questionnaire hosted on survey monkey was used to collect data on demographics, field epidemiology experience, skills application, career development, involvement in international public health work, continuing education, and overall satisfaction with the field epidemiology career.

**Results:**

A total of 974 FETP graduates completed the questionnaire. Most participants were aged 30–39 years, with a higher proportion of advanced graduates aged 40–49 years, and males predominating across all tiers. Graduates across all tiers reported frequent use of field epidemiology skills in their professional roles. International deployment for public health events was reported by 18.4% of intermediate, 13.8% of frontline/basic, and 12.4% of advanced FETP graduates. The impact of FETP on career trajectories and professional development was widely acknowledged. Among advanced FETP graduates, 21.6% earned a master’s degree through FETP, while 20.6% pursued one independently post-graduation. Additionally, 16.6% of advanced FETP graduates completed a diploma through FETP, and 3.4% obtained a PhD afterward. Expanded job responsibilities were reported by 89.4% of intermediate, 86.0% of frontline/basic, and 84.7% of advanced FETP graduates. Many also credited FETP with enhancing job opportunities. Advanced statistics was the most frequently requested topic for further training, cited by 64.9% of advanced, 59.7% of intermediate, and 45.2% of frontline/basic FETP graduates. Satisfaction with salary and benefits was relatively low (44.3%), while satisfaction with co-worker relationships and work-life balance was higher (82 and 65.9%, respectively).

**Conclusion:**

FETP graduates across all tiers demonstrated consistent application of field epidemiology and public health skills in their careers. They reported positive impacts on job responsibilities, educational advancement, and professional growth. Identified continuing education needs—particularly in advanced statistics, emergency management, and data visualization—point to key areas for ongoing development.

## Introduction

The Field Epidemiology Training Program (FETP) is an in-service training designed to strengthen the epidemiologic capacity of staff in ministries of health and national public health institutes. It equips the public health workforce with essential skills in field epidemiology and other core public health competencies. Launched in 1980 with support from the U.S. Centers for Disease Control and Prevention (CDC), FETP is modeled after the Epidemic Intelligence Service (EIS), an applied epidemiology training program established by the CDC in 1951 (1–3). The program is delivered in a mentored and learn-by-doing approach, with emphasis on fieldwork (1).

FETP graduates are public health professionals skilled in disease surveillance, outbreak investigation, public health program development, public health services, and public health emergency response (1). In addition, many graduates serve as mentors or trainers for their FETPs. When health threats emerge, they track, analyze, and contain outbreaks. They provide essential expertise and leadership to ensure timely communication of vital information to health officials and communities, ultimately saving lives and preventing the spread of diseases.

FETP is based on a three-tiered pyramid model that includes basic, intermediate, and advanced training levels (1). FETP of all levels consists of 25% instruction and 75% practical application of the concepts learned to activities performed in the workplace. The basic-level FETP, or frontline FETP, is a three-month training program that focuses on core skills for frontline surveillance. The intermediate-level FETP is a nine- to twelve-month program that builds on these foundations by enhancing competencies in descriptive epidemiology, data analysis, outbreak investigations, and producing reports for external use. The advanced-level FETP is a two-year program that provides comprehensive training in applied epidemiology, enabling trainees to lead analytic outbreak investigations, design and conduct planned studies, evaluate and improve surveillance systems, and contribute to the scientific literature through peer-reviewed publications and conference presentations.

The FETP alumni are actively contributing to public health capacity, responding to emergencies, and advancing scientific knowledge. Over 22,000 FETP alumni worldwide have been trained to detect and respond to public health threats, such as infectious disease outbreaks, chronic illnesses, natural disasters, and humanitarian crises (1). In total, trainees and alumni of Training Programs in Epidemiology and Public Health Interventions Network (TEPHINET) member FETPs have developed, implemented, or evaluated more than 8,680 disease surveillance systems, investigated more than 14,190 outbreaks or acute health events, delivered more than 11,250 oral and poster presentations at scientific conferences, and published more than 3,710 peer-reviewed articles (1).

Although all programs have a standard structure and curriculum, each country tailors the content to fit its specific context. FETP training in the Eastern Mediterranean Region (EMR) integrates e-training methods to reach a broader audience at a lower cost while maintaining effectiveness (4). A study evaluating the FETP in EMR countries found that the advanced FETP graduates in the EMR were well engaged in many field epidemiology activities, with nearly two-thirds of graduates often managing public health surveillance systems and resources, analyzing data, training professionals, and responding to outbreaks (5).

The EMR faces unique public health challenges that heighten the need for skilled epidemiologists. In recent years, many EMR countries have experienced conflicts, humanitarian crises, and climate-driven disasters, all of which have contributed to frequent outbreaks and strained health systems. Against this backdrop, FETP graduates are critical for detecting and responding to priority health threats in the region. Despite global recognition of FETP’s value, existing evaluations have mostly focused on short-term outputs rather than long-term workforce outcomes. Moreover, career trajectories and ongoing training need remain under-studied. Understanding these factors will help optimize the program’s structure, content, and support mechanisms to better serve the workforce and public health systems.

Despite the well-recognized benefits of FETP, limited empirical evidence exists on how participation in the program translates into career advancements, leadership opportunities, and professional development. Furthermore, identifying gaps in continuing education and professional satisfaction will inform program enhancements, ensuring sustained engagement and effectiveness. Therefore, this study aimed to assess the utilization of field epidemiology skills and the perceived confidence of graduates in applying these skills throughout their careers. It also sought to assess the impact of FETP on the professional growth of graduates across different tiers, identify their continuing education needs, and identify areas of dissatisfaction. The findings of this study are expected to provide valuable insights for FETP stakeholders, including program administrators, policymakers, and funding agencies. These insights will contribute to ongoing improvements in the program, ensuring its continued relevance and effectiveness in developing a highly skilled and resilient public health workforce.

## Methods

### Design

A cross-sectional, descriptive study was conducted among graduates from various levels of FETP (frontline/basic, intermediate, advanced) across different countries in the EMR and Bangladesh in 2024. This survey was conducted through a collaborative effort between Training Programs in Epidemiology and Public Health Interventions Network (TEPHINET), the Eastern Mediterranean Public Health Network (EMPHNET), and the US CDC. Eligible participants were all individuals who had completed any level of the FETP (frontline/basic, intermediate, or advanced) in countries where EMPHNET supports the program, including those in the EMR and Bangladesh. Graduates from the inception of each program through the most recent cohorts were included, with no additional inclusion or exclusion criteria beyond program completion. Because the survey link was distributed through multiple channels, including direct emails and onward sharing by national FETP directors, the exact number of graduates who received the invitation could not be determined, and therefore a precise response rate could not be calculated.

### Data collection

The questionnaire was administered in both Arabic and English. The survey was automated through SurveyMonkey, an online survey platform, and then sent to FETP graduates via email. It was also shared with country FETP directors, who were asked to distribute it within their networks. The FETP alumni roster maintained by EMPHNET, which compiles graduates from all program tiers across the EMR and Bangladesh, was used to identify participants. An initial email invitation was sent, followed by multiple weekly reminders. Because the roster is generally comprehensive, respondents represented all program levels and a wide range of graduation years, enhancing the study’s representativeness. Returned surveys were reviewed for consistency, and the SurveyMonkey platform applied skip-logic checks to prevent invalid responses. These quality control measures enhanced the reliability of the analyzed dataset.

### Questionnaire

The questionnaire was divided into several sections, each focusing on a different aspect of the graduate’s career and experiences. The questionnaire included multiple-choice questions, checklists, drop-down lists, and Likert scales (agreement, satisfaction, skills confidence levels, and others). The questionnaire included conditional branching and customized follow-up questions depending on the highest FETP tier obtained and chosen. Questions were also supported by elaborative and instructional text to enhance understanding and improve the flow of responses. Introductory information about the survey and a consent form for participation were provided at the beginning.

The first section included demographic data and general information about the respondents as age, gender, primary nationality, and spoken languages. The second section included information on the FETP training program, including the type of program they completed (frontline/basic, intermediate, or advanced) and the year of graduation. The third section explored graduates’ confidence level in performing the skills learned during the FETP and the current application of these skills in their professional roles. The skills were derived from the CDC competency framework specific to each tier and included public health surveillance, field investigation, epidemiological methods, and scientific communication. Answers included five options: (1) basic knowledge of the skill, but no experience, (2) limited experience with the skill, but still needs help performing it, (3) can perform the skill independently, but still needs occasional help from an expert, (4) can perform this skill without assistance, and (5) expert in this skill and can provide guidance on how to use this skill.

The fourth section included questions on the graduates’ training and education, such as their highest educational attainment and levels of professional experience in different areas. The fifth section delved into more detail about their professional experience in terms of the current employment sector and job title. The sixth section included a question on deployment experience and opportunities, like being deployed to another country to respond to a public health event and taking additional training related to public health emergencies. The seventh section focused on career expectations, including a question on how likely graduates are to recommend pursuing FETP certification.

The final section included two tables assessing graduates’ opinions on the FETP program’s impact on their work and opportunities (Strongly Disagree to Strongly Agree) and the overall satisfaction of graduates with their career in different aspects, such as salary, benefits, work-life balance, and other items (Very dissatisfied to Very satisfied). Responses in this section were restricted to graduates from cohorts directly supported by EMPHNET, ensuring that the feedback was directly relevant to assessing EMPHNET’s role and contributions. This section also included a question on their engagement in learning activities such as conferences, webinars, workshops, or seminars since graduation from FETP.

### Ethical considerations

Ethical approval for the study was obtained from the Institutional Review Board at EMPHNET (IRB Protocol Number: 2024/1/E3). Ethical principles were emphasized throughout the study to ensure that participants’ rights were protected. The survey included an introductory section outlining the associated organizations participating in the survey, the purpose, and the benefits of participation. It also emphasized that responses would be kept confidential and would not be shared with supervisors, the Ministry of Health, or staff affiliated with the FETP. Additionally, contact information was provided in case invitees had any questions or concerns. Participation was voluntary, and respondents were free to exit the survey at any point. Informed consent was obtained from all respondents. All responses were kept anonymous, and data confidentiality was strictly maintained throughout the study phases.

### Statistical analysis

Data were analyzed using IBM SPSS Statistics, version 24. Descriptive statistics, including frequencies and percentages, were used to summarize categorical variables. Missing values were not imputed because the primary aim was to describe graduates’ characteristics and perceptions rather than to generate predictive models or test hypotheses. Consequently, the denominator for percentage calculations varied by variable, and results are reported based solely on available data to ensure transparency about actual response patterns. No inferential statistical tests were performed across FETP tiers or other variables because graduates represented diverse countries, time periods, and contexts, limiting the validity of direct comparisons. Moreover, the primary aim of the study was to provide an overview of graduates’ characteristics, career trajectories, and perceptions rather than to test specific hypotheses.

## Results

### Participants’ characteristics

A total of 974 FETP graduates from the EMR and Bangladesh completed the questionnaire, including 300 from the frontline/basic FETP, 295 from the intermediate FETP, and 379 from the advanced FETP, based on their highest FETP tier. Table 1 shows the demographic and relevant characteristics of participants. Most graduates from all tiers fell within the 30–39 years age group (47.0% in frontline, 52.5% in intermediate, and 39.3% in advanced). The advanced tier had a notably higher proportion of graduates aged 40–49 years (40.9%) compared to intermediate (27.5%) and frontline (30.7%). Males dominated across all FETP levels, making up 62.3% of frontline, 67.8% of intermediate, and 64.9% of advanced FETP graduates. Most frontline graduates (54.7%) completed their training within the last 2 years, compared to 46.1% of intermediate and only 25.1% of advanced FETP graduates.

**Table 1:** Demographic and relevant characteristics of 974 FETP graduates from the Eastern Mediterranean Region by highest FETP tier completed.

Variable	Frontline/basic FETP (*n* = 300)		Intermediate FETP (*n* = 295)		Advanced FETP (*n* = 379)	
	*n*	%	*n*	%	*n*	%
Age (year)						
20–29	23	7.7	23	7.8	7	1.8
30–39	141	47.0	155	52.5	149	39.3
40–49	92	30.7	81	27.5	155	40.9
50–59	41	13.7	36	12.2	52	13.7
60 or older	3	1.0	0	0.0	16	4.2
Gender						
Female	113	37.7	95	32.2	133	35.1
Male	187	62.3	200	67.8	246	64.9
Nationality						
Iraq	95	31.7	97	32.9	46	12.1
Afghanistan	3	1.0	92	31.2	10	2.6
Tunisia	22	7.3	24	8.1	2	0.5
Bangladesh	23	7.7	21	7.1	56	14.8
Egypt	38	12.7	15	5.1	29	7.7
Sudan	6	2.0	13	4.4	8	2.1
Morocco	0	0.0	10	3.4	20	5.3
Jordan	12	4.0	4	1.4	63	16.6
Libya	46	15.3	4	1.4	0	0.0
Yemen	13	4.3	8	2.7	9	2.4
Oman	0	0.0	0	0.0	11	2.9
Pakistan	23	7.7	5	1.7	90	23.7
Saudi Arabia	1	0.3	1	0.3	30	7.9
Others	18	6.0	1	0.3	5	1.3
Languages						
Arabic	231	77.0	175	59.3	210	55.4
English	214	71.3	237	80.3	342	90.2
French	28	9.3	33	11.2	27	7.1
Russian	3	1.0	7	2.4	27	7.1
Spanish	1	0.3	0	0.0	1	0.3
Bengali	12	4.0	12	4.1	26	6.9
Dari	2	0.7	23	7.8	3	0.8
Kurdish	9	3.0	7	2.4	0	0.0
Pashto	3	1.0	27	9.2	7	1.8
Urdu	16	5.3	6	2.0	58	15.3
Persian	1	0.3	8	2.7	2	0.5
Years since graduation						
<2	164	54.7	136	46.1	95	25.1
2–3	91	30.3	100	33.9	67	17.7
>3	45	15.0	59	20.0	217	57.3

### FETP skills: utilization and perceived confidence

#### Frontline FETP

Frontline FETP graduates self-reported frequent application of various public health surveillance skills in their current roles (Table 2). A high percentage indicated using skills such as summarizing surveillance data through tables, graphs, and maps (90.9%), interpreting data to detect potential outbreaks (88.4%), conducting data quality audits at reporting sources (80.7%), and generating basic surveillance reports for internal use (90.5%). Additionally, many graduates reported conducting case investigations (86.2%), assisting in outbreak investigations (90.5%), and developing and delivering presentations on work-related projects to internal audiences (82.5%). Most graduates expressed confidence in their ability to apply these field epidemiology skills, with skill levels ranging from needing occasional assistance to being proficient enough to guide others. In addition to applying the skills acquired through the FETP, graduates of the Frontline level are frequently tasked with responsibilities beyond core epidemiology. These include infection prevention and control (83.5%), community engagement (80.1%), and leadership and management (79.7%). Other commonly utilized skills involve health research (74.7%), monitoring and evaluation (72.4%), and risk communication (71.6%).

**Table 2:** Application of frontline field epidemiology skills and confidence levels among frontline FETP graduates in their current roles (*N* = 275).

Frontline competencies/skills	Using skills in the current job		Level of confidence									
			Basic knowledge, but no experience		Limited experience, but still need help		Can perform the skill, but still needs occasional help		Can perform a skill without assistance		An expert and can provide guidance on how to use this skill	
	*n*	%	*n*	%	*n*	%	*n*	%	*n*	%	*n*	%
Public health surveillance												
Summarize surveillance data using simple tables, graphs, and maps	250	90.9	27	9.8	28	10.2	96	34.9	70	25.5	54	19.6
Interpret surveillance data to identify potential outbreaks	243	88.4	26	9.5	36	13.1	110	40.0	63	22.9	40	14.5
Conduct data quality audits at surveillance reporting sources	222	80.7	34	12.4	34	12.4	87	31.6	77	28.0	43	15.6
Produce simple surveillance reports for internal use	249	90.5	26	9.5	34	12.4	69	25.1	95	34.5	51	18.5
Field investigation												
Conduct case investigations	237	86.2	25	9.1	36	13.1	81	29.5	77	28.0	56	20.4
Assist in outbreak investigations	249	90.5	24	8.7	40	14.5	81	29.5	78	28.4	52	18.9
Develop and deliver oral presentations of work-related projects to an internal audience	227	82.5	25	9.1	42	15.3	71	25.8	87	31.6	50	18.2

#### Intermediate FETP

Intermediate FETP graduates also reported frequent use of key public health surveillance skills in their roles (Table 3). The most commonly applied skills included summarizing surveillance data through tables, graphs, and maps (89.4%) and interpreting surveillance data (87.9%). However, fewer graduates reported evaluating the effectiveness and performance of surveillance systems (66.7%). In field investigations, 82.2% reported conducting outbreak investigations using descriptive epidemiology. While most graduates applied epidemiologic methods in their work, fewer were involved in producing surveillance reports for external distribution (66.7%). Overall, the majority expressed confidence in performing these essential field epidemiology skills. In addition to applying the skills acquired through the FETP, many graduates reported engaging in public health activities such as community engagement (82.7%), leadership and management (86.3%), and infection prevention and control (80.2%). Monitoring and evaluation (79.8%), public health or health research (79.4%), statistical programs (71.8%), and risk communication (67.7%) were also commonly used.

**Table 3:** Application of intermediate field epidemiology skills and confidence levels among intermediate FETP graduates in their current roles (*N* = 264).

Intermediate FETP competencies/skills	Using skills in the current job		Level of confidence									
			Basic knowledge, but no experience		Limited experience, but still need help		Can perform the skill, but still needs occasional help		Can perform a skill without assistance		An expert and can provide guidance on how to use this skill	
	*n*	%	*n*	%	*n*	%	*n*	%	*n*	%	*n*	%
Public health surveillance												
Summarize surveillance data using tables, graphs, and maps	236	89.4	15	5.7	17	6.4	73	27.7	80	30.3	79	29.9
Interpret surveillance data	232	87.9	12	4.5	17	6.4	86	32.6	78	29.5	71	26.9
Produce surveillance reports for external distribution	191	72.3	17	6.4	26	9.8	88	33.3	80	30.3	53	20.1
Evaluate the effectiveness and performance of a surveillance system	176	66.7	18	6.8	33	12.5	85	32.2	83	31.4	45	17.0
Field investigation												
Conduct outbreak investigations using descriptive epidemiology	217	82.2	12	4.5	23	8.7	65	24.6	85	32.2	79	29.9
Epidemiologic methods												
Analyze surveillance and outbreak data using descriptive epidemiology methods	216	81.8	10	3.8	23	8.7	68	25.8	80	30.3	83	31.4
Participate in the planning, implementation, and analysis of cross-sectional studies	206	78.0	15	5.7	33	12.5	85	32.2	82	31.1	49	18.6
Interpret surveillance and descriptive epidemiologic data	215	81.4	14	5.3	18	6.8	87	33.0	75	28.4	70	26.5
Scientific communication												
Write field investigation reports	206	78.0	15	5.7	24	9.1	83	31.4	89	33.7	53	20.1
Contribute to scientific reports	207	78.4	21	8.0	26	9.8	85	32.2	81	30.7	51	19.3
Develop and deliver oral presentations of work-related projects	224	84.8	18	6.8	23	8.7	67	25.4	94	35.6	62	23.5

#### Advanced FETP

Advanced FETP graduates reported high engagement with field epidemiology skills in their professional roles (Table 4). Regarding public health surveillance, 86.8% reported analyzing trends and patterns, while 87.7% reported interpreting surveillance data. Additionally, 80.5% reported producing external surveillance reports, and 80.2% reported developing recommendations to enhance surveillance systems. In field investigations, 81.7% of graduates indicated conducting outbreak investigations using analytic epidemiology. Many also reported applying epidemiologic methods, with 82.3% designing and conducting studies and 82.0% actively analyzing data from these studies. Writing field investigation reports (83.8%) and delivering oral presentations at conferences (77.2%) were frequently reported. Regarding broader public health skills, community engagement (89.2%) and leadership and management (90.1%) were the most frequently utilized among graduates. Other commonly applied skills included public health or health research (87.6%), infection prevention and control (84.2%), and risk communication (83.0%).

**Table 4:** Application of advanced field epidemiology skills and confidence levels among advanced FETP graduates in their current roles (*N* = 333).

Advanced FETP competencies/skills	Using skills in the current job		Level of confidence									
			Basic knowledge, but no experience		Limited experience, but still need help		Can perform the skill, but still needs occasional help		Can perform a skill without assistance		An expert and can provide guidance on how to use this skill	
	*n*	%	*n*	%	*n*	%	*n*	%	*n*	%	*n*	%
Public health surveillance												
Analyze surveillance trends and patterns	289	86.8	11	3.3	7	2.1	84	25.2	79	23.7	152	45.6
Interpret surveillance data	292	87.7	9	2.7	12	3.6	79	23.7	85	25.5	148	44.4
Produce surveillance reports for external distribution	268	80.5	10	3.0	16	4.8	82	24.6	93	27.9	132	39.6
Develop and implement recommendations for improving surveillance systems	267	80.2	8	2.4	18	5.4	80	24.0	109	32.7	118	35.4
Field investigation												
Conduct (lead) outbreak investigations using analytic epidemiology	272	81.7	9	2.7	17	5.1	64	19.2	88	26.4	155	46.5
Epidemiologic methods												
Design, write a protocol for, and conduct planned epidemiologic studies	274	82.3	9	2.7	19	5.7	105	31.5	88	26.4	112	33.6
Analyze data from planned studies using analytical epidemiology methods	273	82.0	12	3.6	20	6.0	105	31.5	109	32.7	87	26.1
Interpret and draw evidence-based conclusions from epidemiologic data	279	83.8	10	3.0	16	4.8	99	29.7	102	30.6	106	31.8
Scientific communication												
Write field investigation reports	279	83.8	9	2.7	18	5.4	56	16.8	113	33.9	137	41.1
Write manuscripts for publication in peer-reviewed journals	229	68.8	20	6.0	30	9.0	114	34.2	91	27.3	78	23.4
Write and submit abstracts to scientific conferences	252	75.7	14	4.2	29	8.7	95	28.5	93	27.9	102	30.6
Develop and deliver an oral presentation at a scientific conference	257	77.2	15	4.5	20	6.0	72	21.6	116	34.8	110	33.0
Develop and deliver a poster presentation at a scientific conference	241	72.4	16	4.8	23	6.9	85	25.5	110	33.0	99	29.7

### Public health emergency training completed by FETP graduates

Table 5 presents an overview of additional public health emergency trainings completed by FETP graduates beyond the core curriculum, categorized by FETP tier. Crisis and Emergency Risk Communication (CERC) training emerged as the most frequently reported, with participation rates of 14.9% among frontline/basic graduates, 27.4% among intermediate graduates, and 23.0% among advanced graduates. Training in Epidemiological Methods in Humanitarian Emergencies (CDC/WHO) followed a similar distribution, with 16.1% of frontline/basic graduates, 19.8% of intermediate, and 18.9% of advanced reporting completion. The Global Outbreak Alert & Response Network (GOARN) training was completed by 12.3% of Frontline/Basic, 12.1% of intermediate graduates, and 19.6% of advanced. The CDC’s Public Health Emergency Management (PHEM) Fellowship was undertaken by 8.8% of frontline/basic graduates, 17.3% of intermediate graduates, and 14.0% of advanced graduates. Meanwhile, participation in the United Nations Disaster Assessment & Coordination (UNDAC) training remained relatively low across all tiers, with only 3.8% of frontline/basic, 3.6% of intermediate graduates, and 5.3% of advanced reporting completion.

**Table 5:** Public health emergency training completed by FETP graduates, by FETP tier.

Public health emergency training	Frontline/basic FETP			Intermediate FETP			Advanced FETP		
	Total	*n*	%	Total	*n*	%	Total	*n*	%
Crisis and Emergency Risk Communication—CERC	262	39	14.9	248	68	27.4	322	74	23.0
Epidemiological Methods in Humanitarian Emergencies (CDC/WHO)	261	42	16.1	247	49	19.8	323	61	18.9
Global Outbreak Alert & Response Network—GOARN (WHO)	260	32	12.3	248	30	12.1	321	63	19.6
Public Health Emergency Management—PHEM—Fellowship (CDC)	261	23	8.8	249	43	17.3	321	45	14.0
United Nations Disaster Assessment & Coordination (UNDAC)	263	10	3.8	250	9	3.6	321	17	5.3

### FETP graduates’ experiences in disease prevention, detection, and response

Table 6 summarizes FETP graduates’ involvement in disease prevention, detection, and response activities, organized by FETP tier. COVID-19 was the most commonly addressed public health issue, with 74.7% of frontline/basic, 81.9% of intermediate, and 84.8% of advanced graduates reporting direct involvement. Participation in diarrheal disease response was reported by 44.4% of frontline/basic, 50.8% of intermediate, and 67.7% of advanced graduates. In cholera response efforts, 35.2% of frontline/basic, 49.2% of intermediate, and 48.8% of advanced graduates were involved. For dengue fever, 26.4% of frontline/basic, 23.8% of intermediate, and 46.3% of advanced graduates reported engagement. In contrast, limited participation was noted in responses to diseases such as Lassa fever, Marburg, and Zika. Notably, no intermediate-level graduates reported involvement in Lassa fever activities. Engagement in Ebola response was minimal across all tiers, with only 5.4% of frontline/basic, 2.0% of intermediate, and 7.8% of advanced graduates reporting experience.

**Table 6:** Experiences of FETP graduates in disease prevention, detection, and response, by FETP tier.

Have worked to prevent, detect, or respond to the following diseases	Frontline/basic FETP			Intermediate FETP			Advanced FETP		
	Total	*n*	%	Total	*n*	%	Total	*n*	%
Chikungunya	262	16	6.1	247	21	8.5	323	42	13.0
Cholera	261	92	35.2	248	122	49.2	322	157	48.8
COVID-19	261	195	74.7	248	203	81.9	322	273	84.8
Dengue fever	261	69	26.4	248	59	23.8	322	149	46.3
Diarrheal diseases	261	116	44.4	248	126	50.8	322	218	67.7
Ebola	259	14	5.4	250	5	2.0	321	25	7.8
Lassa fever	259	7	2.7	250	0	0.0	313	5	1.6
Malaria	260	56	21.5	248	72	29.0	322	108	33.5
Marburg	258	8	3.1	250	1	0.4	318	7	2.2
Measles	261	150	57.5	248	166	66.9	322	197	61.2
Middle east respiratory syndrome (MERS-CoV)	262	34	13.0	248	31	12.5	323	80	24.8
Monkeypox	261	43	16.5	248	27	10.9	322	77	23.9
Other vaccine-preventable diseases	261	73	28.0	248	78	31.5	322	155	48.1
Poliomyelitis	261	88	33.7	248	87	35.1	322	151	46.9
Severe acute respiratory syndrome (SARS-CoV)	261	52	19.9	248	54	21.8	322	98	30.4
Yellow fever	259	14	5.4	250	12	4.8	321	25	7.8
Zika	263	5	1.9	250	5	2.0	324	11	3.4

### Deployment experience and interest in international response opportunities

International deployment for public health events was reported by 13.8% of frontline/basic, 18.4% of intermediate, and 12.4% of advanced graduates. Despite these relatively low deployment rates, there was strong interest across all FETP tiers in joining TEPHINET’s emergency deployment roster for international public health responses. Interest was highest among intermediate graduates (96.3%), followed by advanced graduates (94.3%) and frontline/basic graduates (89.0%). In addition to deployment opportunities, FETP graduates expressed a strong desire to connect with professional colleagues and access resources through TEPHIConnect. Nearly all advanced graduates (98.1%) expressed interest in joining the platform, alongside 96.3% of intermediate and 95.7% of frontline/basic graduates.

### Graduates’ perceived impact of FETP on career advancement and professional growth

The impact of FETP on graduates’ career trajectories and professional growth was widely recognized across all tiers. Among advanced FETP graduates, 21.6% earned a Master’s degree through the FETP, while an additional 20.6% pursued a Master’s degree independently after graduation from FETP. Furthermore, 16.6% of advanced graduates completed a diploma through FETP, and a smaller proportion (3.4%) continued their education after FETP graduation to pursue a PhD. Additionally, 10.3% of graduates continued their education post-FETP, specializing in community medicine. Similarly, 16.6% of intermediate FETP graduates and 13.7% of frontline FETP graduates continued their education and earned a Master’s degree.

The perceived benefits of FETP were consistently high across all tiers, as summarized in Table 7. A significant majority of graduates across all levels attributed expanded job responsibilities to their participation in the program—86.0% of frontline/basic, 89.4% of intermediate, and 84.7% of advanced graduates. Similarly, improved job opportunities were reported by 84.4% of frontline/basic, 85.9% of intermediate, and 84.0% of advanced graduates. Graduates also reported that FETP broadened the scope of their work. This was indicated by 87.2% of frontline/basic, 87.4% of intermediate, and 88.4% of advanced graduates. Access to further education and training opportunities was another key benefit: 90.5% of frontline/basic, 93.9% of intermediate, and 89.9% of advanced graduates acknowledged improved access since completing the program.

**Table 7:** Perceived impact of FETP on career advancement and professional growth among graduates trained under EMPHNET’s direct support.^a^

Dimension	Frontline/basic FETP (*n* = 179)		Intermediate FETP (*n* = 198)		Advanced FETP (*n* = 268)	
	*n*	%	*n*	%	*n*	%
Additional job responsibilities	154	86.0	177	89.4	227	84.7
Benefits, such as health insurance and a retirement plan	114	63.7	133	67.2	150	56.0
Better job opportunities	151	84.4	170	85.9	225	84.0
Broadened scope of work	156	87.2	173	87.4	237	88.4
Education and training opportunities	162	90.5	186	93.9	241	89.9
Independence and control in work-related decision-making	154	86.0	173	87.4	228	85.1
Leadership and management opportunities	155	86.6	173	87.4	230	85.8
Networking opportunities	159	88.8	172	86.9	226	84.3
Professional advancement or promotion	144	80.4	164	82.8	214	79.9
Recognition from co-workers	154	86.0	171	86.4	228	85.1
Relationship with co-workers	157	87.7	172	86.9	230	85.8
Salary increase	91	50.8	96	48.5	145	54.1

a Analysis was limited to graduates who received training under EMPHNET’s direct support.

Increased autonomy and control over work-related decisions was another notable impact, reported by 86.0% of frontline/basic, 87.4% of intermediate, and 85.1% of advanced graduates. Opportunities to take on leadership and management roles were also widely recognized, with 86.6% of frontline/basic, 87.4% of intermediate, and 85.8% of advanced graduates highlighting this benefit. Professional networking emerged as a standout advantage, with 88.8% of frontline/basic, 86.9% of intermediate, and 84.3% of advanced graduates reporting improved professional connections due to their participation in FETP. Similarly, professional advancement and promotions were attributed to the program by 80.4% of frontline/basic, 82.8% of intermediate, and 79.9% of advanced graduates.

Recognition from colleagues due to FETP training was noted by 86.0% of frontline/basic, 86.4% of intermediate, and 85.1% of advanced graduates. Since completing the program, many graduates have engaged in professional development activities, such as attending conferences, webinars, workshops, and seminars. These activities were reported by 61.5% of frontline/basic, 78.8% of intermediate, and 86.9% of advanced graduates.

### Continuing education needs for FETP graduates

Table 8 presents the continuing education needs identified by FETP graduates, categorized by tier. Advanced statistics emerged as the most frequently requested topic, with 64.9% of advanced, 59.7% of intermediate, and 45.2% of frontline/basic graduates indicating its relevance to their work. Emergency management or response training was another top priority, cited by 55.3% of advanced, 49.4% of frontline/basic, and 49.2% of intermediate graduates. Data visualization was also highlighted as an important area, with 51.6% of advanced graduates and 42.5% of both intermediate and frontline/basic graduates recognizing its value. Interest in Geographic Information Systems (GIS) training was strong among advanced (53.7%) and intermediate (42.7%) graduates, though slightly lower among frontline/basic graduates (32.2%). Grant writing was particularly emphasized by advanced graduates (51.6%), with lower demand from intermediate (32.3%) and frontline/basic (22.6%) graduates, reflecting the greater need for proposal development and research funding skills at the advanced level.

**Table 8:** Types of additional continuing education beneficial for FETP graduates, by FETP tier.

Additional continuing education beneficial for FETP graduates	Frontline/basic FETP (*n* = 261)		Intermediate FETP (*n* = 248)		Advanced FETP (*n* = 322)	
	*n*	%	*n*	%	*n*	%
Advanced statistics	118	45.2	148	59.7	209	64.9
Behavioral science	47	18.0	50	20.2	87	27.0
Chronic disease epidemiology	111	42.5	86	34.7	143	44.4
Community engagement	90	34.5	83	33.5	116	36.0
Data visualization	111	42.5	109	44.0	166	51.6
Emergency management or response	129	49.4	122	49.2	178	55.3
Environmental epidemiology	108	41.4	102	41.1	148	46.0
Geographic information systems	84	32.2	106	42.7	173	53.7
Grant writing	59	22.6	80	32.3	166	51.6
Management and leadership	100	38.3	105	42.3	151	46.9
Mentorship	53	20.3	96	38.7	110	34.2
Program monitoring and evaluation	85	32.6	97	39.1	130	40.4
Public health economics	76	29.1	77	31.0	133	41.3
Risk communication	107	41.0	111	44.8	140	43.5
Scientific writing (e.g., abstracts, manuscripts, etc.)	80	30.7	93	37.5	151	46.9
Social epidemiology	80	30.7	74	29.8	93	28.9
Spatial epidemiology	55	21.1	49	19.8	104	32.3
Statistical programs	101	38.7	118	47.6	181	56.2

### Graduates’ satisfaction with their field epidemiology career

Table 9 shows FETP graduates’ satisfaction with various career aspects. Satisfaction with salary adequacy varied across tiers, with 48.5% of advanced graduates and 48.5% of intermediate graduates expressing satisfaction. In contrast, only 33.5% of frontline/basic graduates were satisfied, while the remaining graduates across all tiers expressed dissatisfaction. Recognition from colleagues from other fields was reported by 74.7% of intermediate, 69.3% of frontline/basic, and 67.9% of advanced graduates as a source of satisfaction. Independence and control over work-related decisions were especially valued by intermediate (82.3%), followed by advanced (69.0%) and frontline/basic (67.0%) graduates. Work-life balance was reported by 69.7% of intermediate, 64.9% of advanced, and 63.1% of frontline/basic graduates. Opportunities for advancement, particularly in education and training, were reported by 67.7% of intermediate, 63.1% of advanced, and 61.5% of frontline/basic graduates. Benefits, such as health insurance and retirement plans, were only mentioned by 44.4% of intermediate, 41.8% of advanced, and 38.5% of frontline/basic graduates. Satisfaction related to relationships with co-workers was reported by 84.0% of advanced, 83.3% of intermediate, and 77.7% of frontline/basic graduates. Finally, a majority of respondents reported that they are likely to recommend FETP to others, with 73.0% of advanced, 72.1% of intermediate, and 62.6% of frontline/basic graduates indicating their willingness to endorse the program.

**Table 9:** Satisfaction with field epidemiology careers among graduates trained under EMPHNET’s direct support*.

Satisfaction items	Frontline/basic FETP (*n* = 179)		Intermediate FETP (*n* = 198)		Advanced FETP (*n* = 268)	
	*n*	%	*n*	%	*n*	%
Adequacy of your salary	60	33.5	96	48.5	130	48.5
The level of recognition from co-workers in other fields for the work you do	124	69.3	148	74.7	182	67.9
The degree of independence and control that you have in making decisions related to your work	120	67.0	163	82.3	185	69.0
Work-life balance	113	63.1	138	69.7	174	64.9
Opportunities for advancement, such as education and training opportunities related to your work	110	61.5	134	67.7	169	63.1
Benefits you receive, including the health insurance and retirement plan	69	38.5	88	44.4	112	41.8
Relationship with co-workers	139	77.7	165	83.3	225	84.0

*Analysis was limited to graduates who received training under EMPHNET’s direct support.

## Discussion

This study included more males (65.0%) than females (35.0%). This is consistent with the gender distribution of FETP graduates across most research evaluating FETP programs (6, 7). This disparity reflects the challenging nature of field epidemiology, which often involves conducting outbreak investigations in remote and challenging environments.

In this study, most graduates across the three tiers reported utilizing a variety of public health surveillance skills in their current roles. These skills include summarizing data, interpreting data to identify potential outbreaks, and conducting data quality audits. This aligns with the positive impact of FETPs (7). An evaluation conducted by the CDC, in partnership with the TEPHINET and participating countries, revealed that fellows had opportunities to enhance their surveillance skills and to work with their host country’s surveillance systems (8). The scoping review (7) showed that previous studies identified notable improvements in data quality, reporting practices, and the completeness and timeliness of surveillance data.

One study in the EMR and Bangladesh assessed the impact of advanced FETPs and found that graduates were actively engaged in various field epidemiology activities (5). These activities included managing public health surveillance systems, analyzing surveillance data, and training public health professionals (9). A scoping review indicated that the FETP positively influences the epidemiological skills of trainees, regardless of whether they participated in frontline or advanced programs (7). This study showed that over 80% of frontline, intermediate, and advanced FETP graduates have participated in case and outbreak investigations.

This study revealed that graduates frequently utilize various public health skills in addition to conducting surveillance and outbreak investigations. The most common skills among frontline graduates are infection prevention and control, followed by community engagement. For those in the intermediate program, community engagement was the most utilized skill, followed by leadership, management, and infection prevention. Advanced FETP graduates emphasize community engagement, leadership, public health research, and infection prevention. These results are consistent with other studies (9–11).

The experiences of graduates of FETP in this study underscored their roles in preventing, detecting, and responding to various diseases (12). The contribution of FETP graduates to the COVID-19 epidemic was the most frequently mentioned response and is consistent with reports from FETPs of other regions (13, 14). This study highlighted a wide range of disease-related experiences among FETP graduates across the three tiers, underscoring the critical role these programs play in equipping the public health workforce for diverse health threats. Notably, a significant proportion of advanced FETP graduates reported working on high-priority diseases in the EMR and Bangladesh, such as cholera (48.8%), COVID-19 (84.8%), diarrheal diseases (67.7%), measles (61.2%), and MERS-CoV (24.8%). These findings suggest a strong case for aligning FETP curricula more closely with the regional disease burden and public health priorities. Integrating case studies that reflect actual disease events in the studied countries—particularly on cholera, MERS-CoV, and vaccine-preventable diseases—could enhance the contextual relevance of training and improve preparedness. For frontline and intermediate tiers, where experience with emerging diseases like MERS-CoV, monkeypox, and chikungunya is less common, targeted simulation exercises and scenario-based learning could be especially valuable. Overall, these insights can guide FETP curriculum developers to tailor educational content based on disease-specific exposure patterns, ultimately strengthening the competency of graduates in responding to priority health threats in the region.

This study revealed that a small number of FETP graduates are joining emergency deployment rosters to respond to public health emergencies. Despite these relatively low deployment rates, there was strong interest across all FETP tiers in joining TEPHINET’s emergency deployment roster for international public health responses. Among the various training programs, CERC had the highest participation rates among FETP graduates. This suggests that FETP graduates need to engage more in this training as the number of people affected by complex emergencies has risen sharply in the past decade (15, 16). A study in Sudan assessed the Field Epidemiology Training Program (FETP) during the 2023 armed conflict, highlighting its crucial role in public health emergency response. FETP residents and graduates contributed significantly at various levels during the crisis (17). However, strategies are needed to improve the deployment and retention of FETP residents to ensure their availability in emergencies.

Among the FETP graduates in the current study, 18.4% at the intermediate level, 13.8% at the frontline/basic level, and 12.4% at the advanced level have been deployed internationally for public health events. A strong interest in joining TEPHINET’s emergency deployment roster was expressed by most graduates: 96.3% of intermediate-level, 94.3% of advanced-level, and 89.0% of frontline/basic-level graduates. This widespread interest, along with the extensive participation of FETP graduates in the COVID-19 response, underscores the key and potentially expanding role that FETP graduates can play in regional and global emergency responses. Given this, consideration should be given to including an introduction to CERC and related topics in the FETP curriculum, ensuring that graduates are informed about and have access to CERC resources.

In this research, a significant proportion of advanced FETP graduates reported earning diplomas, Master’s degrees, or PhDs after completing the program. While some of these qualifications may have been pursued through academic pathways affiliated with FETP, it is important to note that the decision to pursue further education likely reflects individual motivation and external opportunities, rather than being solely attributed to FETP participation. The EMR region, through EMPHNET, FETPs, and their partners, could explore strengthening collaborations with academic institutions to complement FETP training. Offering joint degree programs, academic credits, or professional certifications could better prepare graduates for both their routine responsibilities and emergency response roles. Strengthening these partnerships would also create pathways for continued education, such as Master’s degrees, which many graduates consider essential for career advancement, as emphasized in regional and global FETP discussions.

FETP graduates across all tiers expressed a desire for further education in advanced statistics, emergency management and response training, data visualization, and geographic information systems (GIS). Notably, graduates from the advanced level (51.6%) highlighted, as they have in previous studies (18, 19), their interest in training on grant writing.

The research findings indicate that FETP graduates across all levels generally reported positive experiences with the program. Over 80% believed the program enhanced their job responsibilities and career opportunities, with many experiencing expanded roles, increased education and training options, and greater independence in their work. Leadership development was a particularly strong outcome, with over 85% recognizing leadership benefits. Since leadership benefits and workplace recognition were strong aspects of the program, FETP alumni networks and mentorship programs could be expanded to further strengthen professional development and peer support. Graduates expressed satisfaction with their job responsibilities and decision-making autonomy, suggesting that the program effectively prepares participants for real-world public health roles. Additionally, over 60% of graduates at all levels were satisfied with their work-life balance, which is crucial for long-term career sustainability.

Despite these positive aspects, salary satisfaction was significantly lower, with less than 50% of advanced and intermediate graduates and only 33.5% of frontline/basic graduates expressing satisfaction. This suggests that while the program enhances competencies, it may not translate into adequate financial rewards, particularly at the frontline/basic level. Moreover, satisfaction with benefits such as health insurance was low, pointing to potential gaps in financial security and overall job compensation.

A notable strength was the high level of recognition from colleagues, indicating that FETP graduates are valued in their workplaces. However, the concerns over salary and benefits could impact long-term retention and motivation, particularly for those in lower-tier positions. Efforts should be made to advocate for competitive salaries and better benefits to ensure long-term workforce sustainability.

One of the key strengths of this study is its relevance to public health policy and workforce development. The study offers actionable insights for improving public health training programs by identifying skill gaps and the need for ongoing education. One significant limitation of the study is its reliance on self-reported graduate data, which may introduce bias. Participants could overestimate their skills or job satisfaction levels, a limitation common in surveys that could affect the validity of specific responses. Furthermore, the study did not thoroughly examine external factors, such as political instability or challenges within healthcare systems in specific countries, that might have influenced graduates’ experiences or the application of their field epidemiology skills. These contextual factors could significantly impact how effectively graduates utilize their training.

In conclusion, the findings suggest that FETPs play an important role in strengthening health systems and preparing a resilient public health workforce capable of addressing emerging health threats and supporting population health in the EMR and beyond. FETP graduates across all tiers demonstrated frequent application of field epidemiology and public health skills in their professional roles. Graduates from the advanced, intermediate, and frontline/basic programs consistently reported positive career outcomes, including increased job responsibilities, access to educational opportunities, and professional growth. Despite some variations in specific areas of training and deployment experience, graduates perceived that the program contributed to enhancing their leadership, management, and public health expertise. However, continuing education needs such as advanced statistics, emergency management, and data visualization highlight areas for further professional development.

These findings carry important implications for multiple stakeholders. Program managers and educators can use them to refine FETP curricula and continuing education, such as strengthening training in advanced analytics and emergency management. Policymakers should formally recognize FETP graduates as part of the core public health workforce by establishing clear career pathways and incentives to retain their expertise. Funding agencies and partners should prioritize sustained investment in FETPs, including alumni mentoring networks, academic partnerships, and emergency deployment capacities. Future research should longitudinally track alumni to assess their impact on health outcomes and evaluate strategies to enhance retention and leadership development. Additionally, initiatives that promote work–life balance and provide graduates with greater independence in decision-making will improve job satisfaction and prepare them for senior roles. Finally, partnerships with universities to expand opportunities for advanced degrees, such as master’s and PhD programs, will further support academic and career progression.

## Data Availability

The raw data supporting the conclusions of this article will be made available by the authors, without undue reservation.
